# p53 and p21 Status Influences Cellular Response to Metformin in *KRAS*-Mutant HCT116 Colorectal Cancer Cells

**DOI:** 10.3390/cimb48070731

**Published:** 2026-07-17

**Authors:** Asma Saeed, Jamila Hijazi, Zainab Bashir, Pierre Khoueiry, Assaad A. Eid, Nadine Darwiche, Maria Teresa Bengoechea-Alonso, Johan Ericsson, Borbala I. Mifsud, Georges Nemer

**Affiliations:** 1 College of Health and Life Sciences, Hamad Bin Khalifa University, Doha P.O. Box 34110, Qatar; assa43869@hbku.edu.qa (A.S.); zaba29905@hbku.edu.qa (Z.B.); malonso@hbku.edu.qa (M.T.B.-A.); pericsson@hbku.edu.qa (J.E.); bmifsud@hbku.edu.qa (B.I.M.); 2 Department of Biochemistry and Molecular Genetics, Faculty of Medicine, American University of Beirut, Beirut P.O. Box 110236, Lebanon; jmh33@mail.aub.edu (J.H.); pk17@aub.edu.lb (P.K.); nd03@aub.edu.lb (N.D.); 3 Department of Anatomy, Cell Biology, and Physiological Sciences, Faculty of Medicine, American University of Beirut, Beirut P.O. Box 110236, Lebanon; ae49@aub.edu.lb

**Keywords:** metformin, colorectal cancer, *KRAS*, tumor suppressor gene, p53, p21, HCT116, transcriptomic analysis

## Abstract

Colorectal cancer (CRC) remains a leading cause of cancer morbidity worldwide, highlighting the need for improved therapies. Metformin, a widely used antihyperglycemic agent, has gained attention for its potential antitumor properties. In this study, we evaluated the effects of the tumor suppressor genes *TP53* and *CDKN1A* on metformin responsiveness in a *KRAS*-mutant CRC in vitro model using HCT116 cells harboring a G13D mutation in *KRAS*. Using parental (p53^+/+^, p21^+/+^) and isogenic knockout cell lines, we assessed cell-cycle distribution and transcriptomic responses following metformin treatment. In parental wild-type cells, metformin exposure was associated with a dose- and time-dependent reduction in cell viability and an increased proportion of cells in the G0/G1 phase, with significance levels across treatment conditions ranging from *p =* 0.03 to *p* < 0.0001. Loss of p53 or p21 was associated with attenuated cellular responses to metformin, with p21-deficient cells responding primarily at higher doses and prolonged exposure (72 h). Transcriptomic profiling revealed extensive differential gene expression in parental cells (1399 DEGs), compared with more limited responses in p53^−/−^ (270 DEGs) and p21^−/−^ cells (32 DEGs). Differentially expressed genes associated with MAPK signaling (*DUSP5*) and inflammatory regulation (*TNFAIP3*) were observed across genotypes, whereas pathway enrichment of DNA replication and chromatin organization was specific to p53-deficient cells. These findings provide a transcriptomic and phenotypic characterization of genotype-dependent cellular responses to metformin and establish a basis for future mechanistic and functional validation studies.

## 1. Introduction

Colorectal cancer (CRC) is the third most commonly diagnosed cancer worldwide, accounting for 10% of all cancer diagnoses, with approximately 1.9 million new cases reported annually [1]. It is also the second leading cause of cancer-related mortality, with approximately 900,000 deaths (9.3% of all cancer-related deaths) reported in 2022 [1]. The global burden of CRC is expected to increase, with projections estimating 3.2 million new cases and 1.6 million CRC-related deaths by 2040 [2]. Colorectal cancer arises from sporadic mutations in the colorectal mucosa, leading to tumor formation in the large bowel and rectum [3,4,5]. Its carcinogenesis is a multistep process driven by mutations in tumor suppressor genes, oncogenes, and epigenetic alterations such as DNA methylation [6,7]. The initiation of CRC is marked by increased genetic alterations in the adenomatous polyposis coli (*APC*) tumor suppressor gene, leading to epithelial hyperproliferation, and subsequent polyp formation [8]. Mutations in the Kirsten rat sarcoma (*KRAS*) proto-oncogene and the tumor suppressor gene tumor protein 53 (*TP53*), drive progression toward advanced-stage CRC [8]. CRC stage subsequently influences the available treatment options [9].

Metformin is a widely used antidiabetic drug that has gained considerable attention for its potential anticancer properties. Although, it is primarily prescribed to lower glucose levels in patients with diabetes, accumulating research suggests that metformin may also affect cancer-related pathways [10]. By reducing insulin levels and improving glycemic control, metformin can decrease the availability of insulin-like growth factor 1 (IGF-1), a key driver of cancer progression [11], while simultaneously inducing energetic stress in cells. In addition, metformin activates AMP-activated protein kinase (AMPK), a metabolic regulator that suppresses anabolic processes and inhibits the mammalian target of the rapamycin (mTOR) pathway associated with tumor growth [12]. Elevated mTOR activity is common in many cancers, and metformin’s ability to activate AMPK contributes to its tumor-suppressive effects [12].

Studies indicate that IGF-1, DNA damage, hyperglycemia, hyperinsulinemia, obesity, and inflammatory factors may contribute to the increased cancer risk associated with diabetes [13]. This association is further supported by the fact that diabetes and cancer share common risk factors, including diet, sex, age, and smoking, as well as dysregulation of the IGF pathway, which plays a crucial role in the development of both conditions [14]. Consistent with these observations, epidemiological studies have reported a 1.41 times higher cancer mortality rate among diabetic patients than among non-diabetic individuals [15]. Clinical and epidemiological evidence also suggests that metformin use is associated with a 30–50% reduction in cancer incidence, particularly in hepatocellular carcinoma [16], pancreatic cancer [17], and colon cancer [18]. Additionally, randomized clinical trials revealed a 40% reduction in abnormal colorectal polyps among metformin users [19]. At the molecular level, metformin has been reported to influence multiple hallmarks of cancer [20], including cancer stem cells (CSCs) [21,22], cell-cycle regulation [23,24,25,26], migration [27], invasion [27], metastasis [28], apoptosis [29,30], metabolism [31,32,33], and immune response modulation [34,35]. Despite these promising findings, the molecular mechanisms underlying metformin’s anticancer activity remain incompletely understood, highlighting the need for further investigation to optimize its application in oncology [20]. Such studies are expected to improve our understanding of metformin’s potential role in cancer treatment.

*KRAS* is among the most frequently mutated proto-oncogenes in CRC, with approximately 40% of CRC patients carrying an activating missense mutation in this gene [36]. CRC patients with *KRAS* mutations typically have a poorer prognosis than those with wild-type *KRAS*, particularly in cases of metastatic disease [37,38]. Abnormal activation of the *KRAS* pathway disrupts upstream signal regulation, leading to resistance against receptor tyrosine kinase (RTK) inhibitors, such as cetuximab and panitumumab, which target the epidermal growth factor receptor (EGFR), in individuals with *KRAS*-mutant CRC [39,40]. The high affinity of *KRAS* for GTP, along with the absence of an ideal small-molecule binding pocket, complicates the development of specific competitive inhibitors to inhibit *KRAS*-driven oncogenesis [36]. Consequently, *KRAS* was historically regarded as “undruggable” [36]. However, recent advances have led to FDA approval of *KRAS* G12C-specific inhibitors, sotorasib and adagrasib, for metastatic CRC [41,42]. These agents are restricted to the G12C variant, which represents only a small subset of *KRAS*-mutant CRC cases with a prevalence of 3–4% [42]. Most KRAS-mutant CRC cases, including those harboring the G13D mutation, remain without approved targeted therapies, highlighting the continued need for alternative therapeutic strategies.

Previous studies have demonstrated that metformin exerts selective antitumor effects in p53-deficient CRC cells by inducing apoptosis, impairing their ability to adapt metabolically to energetic stress [43]. However, other studies indicate that metformin’s antiproliferative actions in CRC cells can be largely cytostatic, driven by AMPK activation, increased ROS production, and cell-cycle arrest without inducing apoptosis [44]. More recently, integrated transcriptomic analyses have revealed that metformin alters microRNA networks to target genes within PI3K-Akt and MAPK/ERK pathways, thereby regulating CRC cell proliferation at the post-transcriptional level [45]. Despite these insights, the role of critical tumor suppressors such as p21 in mediating or modulating metformin’s effects remains unexplored, particularly in *KRAS*-mutant CRC. To address this gap, we evaluated the phenotypic and transcriptomic responses of HCT116-p53^−/−^ and p21^−/−^ cells to metformin. Our findings suggest that loss of either *TP53* or *CDKN1A* is associated with reduced metformin responsiveness. These observations provide a basis for future studies to investigate genotype-based stratification in metformin repurposing trials for CRC. Thus, in this study, the colorectal cancer cell line HCT116, harboring a *KRAS* G13D mutation, along with its p53 (HCT116-p53^−/−^) and p21 (HCT116-p21^−/−^) knockout derivatives, was used to investigate the effect of tumor suppressor status on metformin responsiveness.

## 2. Materials and Methods

### 2.1. Cell Culture

The HCT116 human colorectal carcinoma cell line was originally purchased from the American Type Culture Collection (ATCC, Manassas, VA, USA) and provided by the laboratory of Dr. Johan Ericsson (Hamad Bin Khalifa University). The p53^−/−^ and p21^−/−^ knockout derivative cell lines of HCT116 were obtained from the laboratory of Dr. Nadine Darwiche, having been kindly provided by Dr. Carlos Maria Galmarini (PharmaMar, Madrid, Spain). These knockout cell lines were generated and characterized by targeted homologous recombination in HCT116 cells by Dr. Bert Vogelstein and colleagues [46,47]. All cell lines were cultured in Dulbecco’s Modified Eagle Medium (DMEM) supplemented with 10% Fetal Bovine Serum, 1% penicillin-streptomycin, 1% L-glutamine and 1% sodium pyruvate (all obtained from Gibco, Thermo Fisher Scientific, Waltham, MA, USA) and maintained at 37 °C in an incubator supplied with 5% carbon dioxide (CO_2_).

### 2.2. Western Blot Validation of Knockouts

To confirm the knockout status of *TP53* and *CDKN1A* in the HCT116 derivative cell lines, protein expression was assessed by Western blot analysis. HCT116 wild-type, HCT116-p53^−/−^ and HCT116-p21^−/−^ cells were cultured under standard conditions for 24 h and harvested at approximately 80% confluency. All steps for protein extraction were performed on ice or at 4 °C using pre-chilled buffers. Total protein was extracted using RIPA lysis buffer (Thermo Fisher Scientific, Waltham, MA, USA), supplemented before use with 1 µL of 1 M dithiothreitol (DTT), 1 µL of 0.5 M phenylmethylsulfonyl fluoride (PMSF), and 10 µL protease inhibitor cocktail, per 1 mL of RIPA buffer to prepare a complete lysis buffer solution (all obtained from Thermo Fisher Scientific, Waltham, MA, USA). Sample preparation for gel electrophoresis involved mixing 20 µL of the lysate with 10 µL of 5× Laemmli loading buffer (4% SDS, 20% Glycerol, 125 mM Tris-HCl (pH 6.8), 10% β-mercaptoethanol and 0.1% bromophenol blue). Before performing electrophoresis, the lysates (20 µL per sample) were heated at 95 °C for 5 min and 15 µL per lane of each protein sample was loaded into the 10-well pre-cast 10% polyacrylamide gel (Novex WedgeWell Tris-Glycine Gel, Invitrogen, Thermo Fisher Scientific, Waltham, MA, USA).

Electrophoresis was performed in 1× running buffer composed of 25 mM Tris Base, 190 mM glycine and 0.1% SDS. Protein molecular weight marker (Bio-Rad Laboratories, Hercules, CA, USA) was loaded (5 µL) alongside the samples for size reference. Following electrophoresis, proteins were transferred onto nitrocellulose membranes using the wet transfer method. Membranes were blocked in 5% bovine serum albumin (BSA, Thermo Fisher Scientific, Waltham, MA, USA) prepared in TBST for 1 h at room temperature on a rocking platform. Primary antibodies were diluted in 5% milk prepared in TBST and membranes were incubated overnight at 4 °C in sealed incubation bags on a tube-rolling platform to ensure uniform antibody exposure. Primary antibodies used included phospho-p53 (Ser15) rabbit monoclonal antibody #9282 (Cell Signaling Technology, Danvers, MA, USA) at 1:1000 dilution, p21 Waf1/Cip1 (12D1) rabbit monoclonal antibody #2947 (Cell Signaling Technology, Danvers, MA, USA) at 1:2000 dilution and β-actin mouse monoclonal (AC-15) antibody A5441 (Sigma-Aldrich, St. Louis, MO, USA) at 1:3000 dilution.

After primary antibody incubation, membranes were washed five times for 5 min each, with TBST on a rocking platform. Membranes were then incubated with horseradish peroxidase (HRP)-conjugated secondary antibodies diluted in 5% milk-TBST for 1 h at room temperature, followed by five 5 min washes with TBST. Protein bands were then detected using SuperSignal West Pico PLUS enhanced chemiluminescence (ECL) substrate (Thermo Fisher Scientific, Waltham, MA, USA), prepared according to manufacturer’s instructions by mixing the reagents at a 1:1 ratio. Membranes were incubated briefly in ECL substrate, excess reagent was removed, and signals were visualized using an Invitrogen iBright CL1500 imaging system (Thermo Fisher Scientific, Waltham, MA, USA). Following imaging, membranes were washed (five times for 5 min) and stored submerged in TBST for subsequent use.

### 2.3. Drug-Dose and Time-Course Treatment

A dose- and time-course experiment was conducted to determine the optimal concentration and treatment duration of metformin hydrochloride, AMPK activator (Abcam, Cambridge, UK). A range of metformin concentrations (0.5, 1, 2, 4, and 8 mM) was selected for the study, consistent with concentration ranges previously used to evaluate dose- and time-dependent effects of metformin on HCT116 [44,48]. Although these concentrations exceed the median therapeutic plasma concentration of metformin in patients (approximately 330 µM at a standard dose of 1 g/day), in vitro studies using HCT116 cells and their isogenic p53^−/−^ derivative have similarly used millimolar concentrations (e.g., 5 mM) to observe measurable cellular response [44]. This difference reflects the distinction between acute in vitro exposure and the pharmacokinetics of chronic in vivo dosing, which involves tissue accumulation in sites like the intestinal mucosa and liver [44]. Two different treatment durations were chosen for time-course analysis: 24 and 48 h. If no response was observed at 24 or 48 h, the treatment duration was extended to 72 h. Trypan blue staining (Gibco, Thermo Fisher Scientific, Waltham, MA, USA) was used to assess the preliminary drug effect through live cell counting. All experiments were performed in duplicate.

### 2.4. MTT Cell Viability Assay

The MTT (3-(4,5-dimethylthiazol-2-yl)-2,5-diphenyltetrazolium bromide) assay is a colorimetric method used to assess cellular metabolic activity as an indicator of cell viability. Cells were seeded in 96-well plates at a density of approximately 5000 cells per well. After 24 h of incubation, the cells were treated with various concentrations of metformin (1, 2, 5, and 10 mM), with six replicates per condition. This assay was performed alongside cell-cycle analysis to identify appropriate metformin treatment conditions for downstream transcriptomic analyses. Therefore, a slightly broader concentration range (1–10 mM) was evaluated, consistent with previous studies in HCT116 cells, for the reasons outlined earlier [44,48]. Three treatment conditions were evaluated: 24 h of metformin exposure, 48 h of continuous exposure and a replenishment condition in which after 24 h of the drug treatment, the metformin-infused media was replaced with fresh metformin-containing media, followed by an additional 24 h of incubation, totaling a 48 h incubation period. Following treatment, cells were incubated with MTT solution (Abcam catalog# ab211091, Cambridge, UK) at a final concentration of 0.5 mg/mL in phosphate-buffered saline (PBS, Gibco, Thermo Fisher Scientific, Waltham, MA, USA) for 3 h at 37 °C. To terminate the reaction, 100 µL of MTT stop solution (Abcam catalog# ab211091, Cambridge, UK) was added to each well and the plates were incubated for an additional 1 h. The absorbance was measured at 595 nm using a Multiskan EX reader (Thermo Fischer Scientific, Waltham, MA, USA). Cell viability was calculated using the formula: cell viability (%) = (mean experimental absorbance/mean control absorbance) × 100%.

### 2.5. Flow Cytometry for Cell-Cycle Analysis

Flow cytometric analysis was performed to assess metformin-associated changes in cell-cycle distribution and to support selection of treatment conditions for downstream analysis. Cells were seeded in a 6-well plate at a density of 500,000 cells per well. After 24 h of incubation, the cells were treated with varying concentrations of metformin (0.5, 1, 2, 4, and 8 mM) for 24, 48 or 72 h. Each experimental condition was performed with two independent biological replicates. Media from each well was collected in a separate 50 mL tube after the treatments. The adherent cells were harvested with trypsin and mixed with their corresponding media suspension before being centrifuged for 2 min at 900 rpm. The cells were washed with PBS, resuspended in cold 70% ethanol, and stored at −20 °C for overnight fixation. The fixed cells were then stained with propidium iodide (PI) solution (50 µg/mL PI diluted in PBS and 25 µg/mL RNase A; Thermo Fisher Scientific, Waltham, MA, USA). Cells were incubated in the dark with PI for 2 h at room temperature. BD Accuri C6 Flow Cytometer (BD Biosciences, San Jose, CA, USA) was used with appropriate gating strategies to exclude debris, doublets, with a minimum of 10,000 events collected per sample for analysis and instrument settings were optimized using unstained controls to establish background fluorescence and ensure accurate PI signal detection. Since minimal changes in cell-cycle distribution were observed in HCT116-p21^−/−^ following 24 and 48 h of treatment, an additional 72 h treatment condition was included for this cell line. Graphs were plotted using R, Student’s *t*-tests were performed in Excel and ANOVA was conducted using GraphPad Prism (11.0.2).

### 2.6. RNA Extraction

Based on the MTT and cell-cycle analyses, 4 mM metformin elicited substantial cellular responses that were comparable to those observed at highest tested concentrations (8, 10 mM) and greater than those observed at lower concentrations. Therefore, treatment with 4 mM metformin for 48 h was selected for transcriptomic profiling. Cells were seeded in 6-well plates at a density of 500,000 cells per well, with three biological replicates of both untreated and metformin-treated samples. RNA was extracted using TRIzol according to Invitrogen’s instructions (Thermo Fisher Scientific, Waltham, MA, USA). RNA pellets were maintained in 75% ethanol to preserve sample stability during shipment to Macrogen (Seoul, Republic of Korea) for whole transcriptome sequencing.

### 2.7. RNA-Sequencing and Pathway Enrichment Analysis

Total RNA isolated from metformin-treated (4 mM for 48 h) and untreated HCT116 wild-type, HCT116-p53^−/−^ and HCT116-p21^−/−^ cell lines (*n* = 2 per condition) was subjected to paired-end sequencing on the Illumina NovaSeq platform (San Diego, CA, USA) following library preparation using the TruSeq standard total RNA library prep globin kit (Illumina, San Diego, CA, USA). Raw sequencing reads were processed using CLC Genomics Workbench version 20.0.4 (QIAGEN Digital Insights, Aarhus, Denmark), including quality control assessment (FastQC), adapter trimming, and read alignment to the reference genome (hg19) to generate read count data. Differential gene expression analysis was performed using iDEP.96 (integrated Differential Expression and Pathway analysis), a web-based application that provides a list of differentially expressed genes (DEGs) as output data [49]. Differentially expressed genes were defined using the default iDEP.96 significance threshold of a false discovery rate (FDR)-adjusted *p*-value < 0.05 and an absolute log2 fold change ≥ 1. Transcriptomic data visualization included k-means clustering to generate heatmaps of DEGs, as well as principal component analysis (PCA) and hierarchical clustering to assess sample relationships and treatment effects. Overlapping DEGs between cell lines were identified using Venny 2.1.0. Functional enrichment analysis of DEGs was conducted using ShinyGO 0.80 to identify significantly enriched Gene Ontology (GO) biological processes and Kyoto Encyclopedia of Genes and Genomes (KEGG) pathways (a database providing functional information for systemic gene function analysis). Data visualization and graphical representations were generated using GraphPad Prism (11.0.2) and ShinyGO (0.85.1).

### 2.8. Quantitative Real-Time PCR Validation

To validate selected differentially expressed genes identified by RNA sequencing, quantitative real-time PCR (RT-qPCR) was performed using the third biological replicate of RNA isolated from HCT116 wild-type, p53^−/−^, and p21^−/−^ cells following treatment with 4 mM metformin for 48 h and their corresponding untreated controls. The cDNA was synthesized from 2 µg of RNA using the High-Capacity cDNA Reverse Transcription kit (catalog# 4374966, Thermo Fisher Scientific, Waltham, MA, USA) according to the manufacturer’s instructions. RT-qPCR reactions were performed using PowerUp™ SYBR™ Green Master Mix (Applied Biosystems, Thermo Fisher Scientific, Waltham, MA, USA) on a QuantStudio™ Real-Time PCR System (Applied Biosystems, Thermo Fisher Scientific, Waltham, MA, USA). Gene-specific primers were used to assess the expression of *DUSP5*, *FGD6*, and *FAM111A*, while *ACTB* was used as an endogenous reference gene. Relative gene expression was calculated using the comparative Ct (2^−ΔΔCt^) method. For each genotype, expression levels in metformin-treated cells were normalized to the corresponding untreated control samples. All samples were analyzed in technical duplicate, and data are presented as mean ± SD.

### 2.9. Statistical Analysis

Statistical analyses were performed using GraphPad Prism. Two-way analysis of variance (ANOVA) followed by Tukey’s multiple comparison test was used to assess differences between treatment concentrations and incubation times. Student’s *t*-tests were used for pairwise comparisons where appropriate. A *p*-value < 0.05 was considered statistically significant. Both R (4.4.2) and GraphPad Prism were used for data visualization.

## 3. Results

### 3.1. Metformin Affects Cell Viability in the CRC Cell Line HCT116: A Time- and Dose-Dependent Analysis

**Figure 1:**
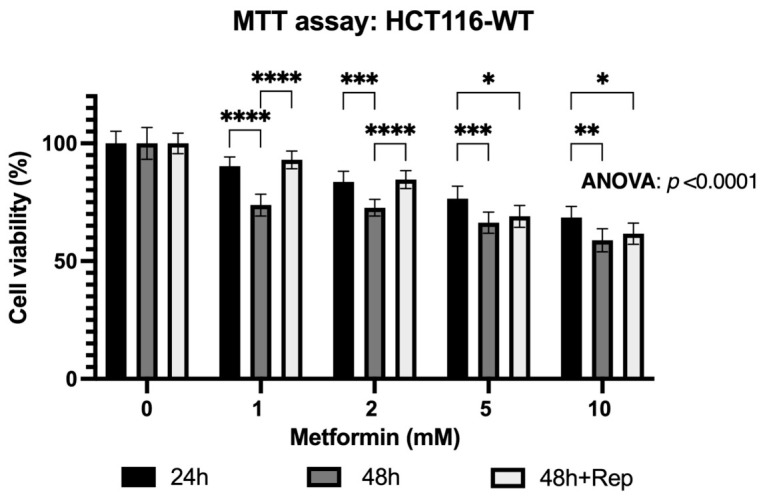
Comparison of HCT116 wild-type cell viability following 24 h metformin treatment, 48 h continuous metformin treatment, and 48 h treatment with metformin replenishment at 24 h (24 h initial treatment followed by replacement with fresh metformin-containing medium and an additional 24 h incubation, totaling 48 h). Cell viability is expressed as a percentage relative to the corresponding untreated control. Two-way ANOVA followed by Tukey’s multiple comparison test was performed using GraphPad Prism (n = 6). Statistical comparisons shown indicate differences between treatment durations at the same concentration. * *p* = 0.0332; ** *p* = 0.0021; *** *p* = 0.0002; **** *p* < 0.0001.

To delineate the effects of metformin on CRC cell viability, MTT assays were performed in HCT116 wild-type cells. Our data demonstrated a dose- and time-dependent reduction in HCT116 cell viability upon metformin exposure, evidenced by a progressive decline in cell viability with increasing metformin concentrations and extended incubation periods (24 h and 48 h, Figure 1). No significant differences were observed between the 48 h continuous exposure and metformin replenishment conditions (Figure 1). The two-way ANOVA confirmed statistically significant effects of both metformin concentration and treatment duration (*p* < 0.0001), as well as a significant interaction between these factors, indicating that the effect of metformin on cell viability varied according to treatment duration. These findings suggest that prolonged exposure was associated with greater reductions in viability, whereas replenishment of metformin after 24 h did not result in substantial further reductions in viability in HCT116-WT cells (Figure 1).

### 3.2. Metformin Is Associated with Changes in Cell-Cycle Distributions in HCT116-WT Cells

**Figure 2:**
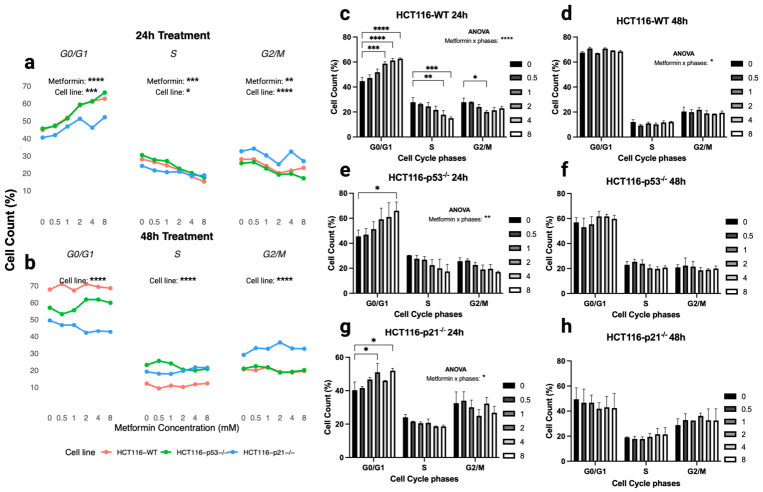
Cell-cycle analysis by propidium iodide staining in HCT116-WT, HCT116-p53^−/−^, and HCT116-p21^−/−^ cells treated with 0–8 mM metformin for 24 h or 48 h. Panels (**a**,**b**) summarize the distribution of cells in G0/G1, S, and G2/M phases across the three cell lines following 24 h and 48 h treatment, respectively. In panels (**a**,**b**), red, green, and blue lines represent HCT116-WT, HCT116-p53^−/−^, and HCT116-p21^−/−^ cells, respectively. Panels (**c**–**h**) show dose-dependent changes in cell-cycle distribution within each cell line and time point: HCT116-WT at 24 h (**c**), HCT116-WT at 48 h (**d**), HCT116-p53^−/−^ at 24 h (**e**), HCT116-p53^−/−^ at 48 h (**f**), HCT116-p21^−/−^ at 24 h (**g**), and HCT116-p21^−/−^ at 48 h (**h**). For panels (**a**,**b**), significance annotations indicate statistically significant main effects identified by two-way ANOVA (cell line and metformin concentration). For panels (**c**–**h**), statistical comparisons were performed within each cell line and time point relative to the untreated 0 mM control using Tukey’s multiple comparison test. Two-way ANOVA followed by Tukey’s multiple comparison test was performed using GraphPad Prism (n = 2 biological replicates). Statistical significance is indicated as follows: * *p* = 0.0332, ** *p* = 0.0021, *** *p* = 0.0002, and **** *p* < 0.0001.

We examined the effects of metformin on cell-cycle distribution using flow cytometry with propidium iodide staining (Table S1, Figure 2). At the 24 h time-point, both wild-type and p53^−/−^ cells showed a concentration-dependent increase in the proportion of cells in G0/G1 accompanied by corresponding decrease in the S and G2/M phases (Figure 2a,c,e). Consistent with these observations, two-way ANOVA indicated that at 24 h, both metformin concentration and cell line (Figure 2a; Table S1) significantly affected the G0/G1, S, and G2/M distributions (ranging from *p* = 0.03 to *p* < 0.0001; Table S1), while interaction terms were not significant. Interestingly, untreated p21^−/−^ cells displayed a higher proportion of cells in G2/M phase compared to other cell lines (Figure 2a,c,e,g), suggesting differences in baseline cell-cycle distribution associated with p21 deficiency. At the 48 h time-point, a high percentage of wild-type cells remained in G0/G1, with minimal variations across metformin concentrations (Figure 2b,d; Table S1). HCT116-p53^−/−^ cells exhibited a modest increase in the proportion of cells in G0/G1 following treatment (Figure 2b,f). Approximately 50% of p21^−/−^ cells were in G0/G1 at 48 h, and this proportion decreased in response to increasing metformin concentrations (Figure 2b,h). At this point, two-way ANOVA indicated that only the cell line factor remained significant across all cell-cycle phases (*p* < 0.0001; Table S1), whereas metformin concentration was no longer significant (Figure 2b), suggesting that differences among the cell lines contributed more strongly to cell-cycle distributions than treatment concentration after prolonged incubation.

Since the impact of metformin on the cell cycle of p21^−/−^ cells was relatively limited at 24 and 48 h, the treatment was extended to 72 h (Table S1). As seen in Figure S1, the percentage of cells in G0/G1 was significantly decreased in response to increasing metformin concentrations (*p* = 0.0332; 2 mM, *p* < 0.0001; 4 mM and *p* = 0.0002; 8 mM; Table S1). Correspondingly, the proportion of cells in G2/M increased significantly (*p* = 0.0021; 2 mM, *p* = 0.0002; 4 mM and *p* = 0.0021; 8 mM; Table S1) at 72 h (Figure S1d; Table S1). The two-way ANOVA across the 24 h, 48 h, and 72 h conditions identified significant interaction effects for G0/G1 and G2/M (Figure 2a,b and Figure S1; Table S1), indicating that the effect of metformin on HCT116-p21^−/−^ cell’s cell-cycle distribution varied according to treatment durations.

In summary, metformin treatment was associated with changes in cell-cycle distributions, with wild-type cells showing a greater increase in proportion of cells in G0/G1 than the p53^−/−^ and p21^−/−^ derivatives. In contrast, changes in cell-cycle distributions in p21-deficient cells became more apparent following prolonged treatment, including an increase in the proportion of cells in the G2/M phase. These observations indicate altered cell-cycle distributions in the absence of p21, which is known to be a key mediator of p53-dependent G1/G2 arrest following DNA damage [50,51]. However, given the variability in the knockout cell responses and the limited sample size, these observations should be interpreted with caution and require further validation.

### 3.3. Western Blot Validation of p53 and p21 Knockout Phenotypes in the Derivative Cell Lines

To confirm the stability of the cell models used in this study, phospho-p53 (Ser15) and p21 protein expression were assessed by Western blot analysis under untreated conditions (Figure S2). Phospho-p53 (Ser15) expression was detected in HCT116 wild-type and HCT116-p21^−/−^ cells but not in HCT116-p53^−/−^ cells (Figure S2a). Similarly, p21 expression was observed in wild-type and p53^−/−^ cells but not in p21^−/−^ cells (Figure S2b). These results are consistent with the reported characteristics of the knockout cell lines [46,47] and support the maintenance and validity of the expected knockout phenotypes throughout the study.

### 3.4. Transcriptomic Profiling Reveals Attenuated Gene Expression Responses in p53 and p21 Knockouts Compared to HCT116 Wild-Type

#### 3.4.1. Principal Component Analysis (PCA)

**Figure 3:**
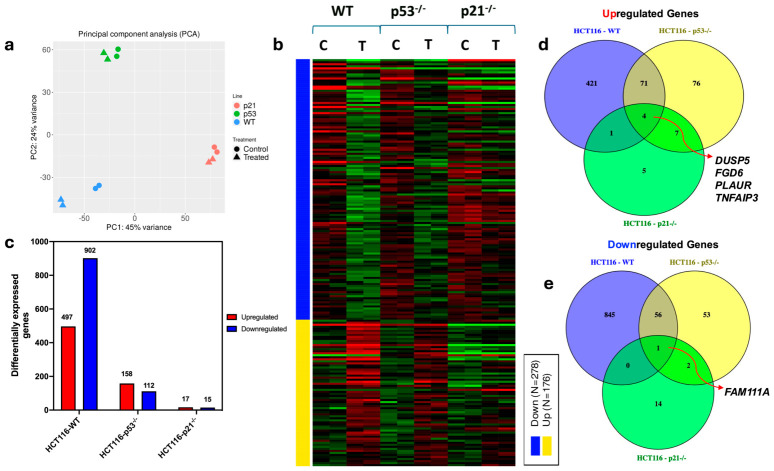
Transcriptomic analysis of metformin-treated HCT116 wild-type (WT), p53^−/−^ and p21^−/−^ cells. (**a**) Principal component analysis (PCA) showing clustering by genotype and treatment status after 48 h of 4 mM metformin exposure. (**b**) Heatmap of differentially expressed genes identified following treatment at a false discovery rate (FDR) cutoff of 0.05, red indicating higher expression and green indicating lower expression. (**c**) Bar plot showing the number of up- and down-regulated DEGs across the three cell lines. (**d**) Venn diagram showing the overlap in the upregulated DEGs among the three cell lines and the four genes upregulated across them. (**e**) Venn diagram showing the overlap in downregulated DEGs and the gene commonly downregulated across the three cell lines.

Consistent with the preliminary findings, the overall transcriptomic changes showed a similar pattern. The PCA of the metformin-treated (4 mM for 48 h) and untreated HCT116 wild-type and its knockout derivatives indicated that the three cell lines exhibited distinct transcriptional profile from one another (Figure 3a). Each cell line formed a separate cluster, indicating unique gene expression patterns driven by their specific genetic alterations. The wild-type cells displayed the most distinct separation between their treated and untreated clusters, indicating a larger transcriptional response to metformin compared to its knockout derivatives (Figure 3a).

#### 3.4.2. Differential Expression Analysis

The distinct clustering observed in the PCA was consistent with differences in baseline gene expression profiles between HCT116-WT and the knockout cell lines. Differential expression analysis of untreated cells relative to the wild-type (Figure S3) identified 1112 downregulated genes and 384 upregulated genes in p53^−/−^ cells, while the p21^−/−^ cells exhibited 1288 downregulated and 1011 genes upregulated (Figure S3). The larger number of differentially expressed genes observed in p21^−/−^ cells suggests a greater degree of transcriptional divergence from parental the cell line. These results suggest loss of p53 and p21 is associated with substantial alterations in baseline gene expression, which may contribute to differences in cellular responses to metformin observed across the three genotypes. Differential expression analysis (Figure 3b,c; Figure S5) following 48 h treatment (4 mM) revealed that HCT116-WT cells exhibited the largest transcriptional response, with 902 downregulated and 497 upregulated genes relative to the untreated control (Figure 3b,c). Notably, *CDKN1A*, a key regulator of the G1-checkpoint and transcriptional target of p53, was modestly but significantly upregulated in metformin-treated wild-type cells (log2 fold change = 1.24, adjusted *p* = 1.17 × 10^−21^), whereas no significant regulation was observed in the p53- or p21-deficient cells (Table S2). In contrast, the HCT116-p53^−/−^ cell line showed 112 downregulated and 158 upregulated genes following treatment, while the HCT116-p21^−/−^ cell line exhibited the smallest transcriptional response, with only 15 downregulated and 17 upregulated genes (Figure 3c). Given the limited number of biological replicates (n = 2), these findings should be interpreted as exploratory and require independent validation.

#### 3.4.3. Overlapping DEGs Among the HCT116 Wild-Type and Its Knockout Derivatives

The Venn diagram (Figure 3d,e) illustrates the overlapping differentially expressed genes (DEGs), both upregulated (Figure 3d) and downregulated (Figure 3e), following metformin treatment of the three cell lines. These shared genes may represent candidate genes involved in cellular response to metformin, as their expression was consistently altered across the three genotypes following treatment. Notably, four genes (*DUSP5*, *FGD6*, *PLAUR*, and *TNFAIP3*) were consistently upregulated across all three cell lines after 48 h of metformin treatment at a dose of 4 mM (Figure 3d). In contrast, only one gene, *FAM111A*, was commonly down-regulated (Figure 3e). To support the RNA-seq findings, RT-qPCR was performed for selected shared DEGs. Consistent with the transcriptomic analysis, *DUSP5* and *FGD6* showed increased expression following metformin treatment in wild-type, p53^−/−^, and p21^−/−^ cells, whereas *FAM111A* expression was reduced across all three genotypes (Figure S4). These results support the RNA-seq data and suggest a shared molecular response to metformin across the three cell lines.

### 3.5. KEGG Pathway Analysis of DEGs Revealed Enrichment of Cellular Senescence and Apoptosis-Related Pathways in Metformin-Treated Wild-Type and p53-Deficient Cells

**Figure 4:**
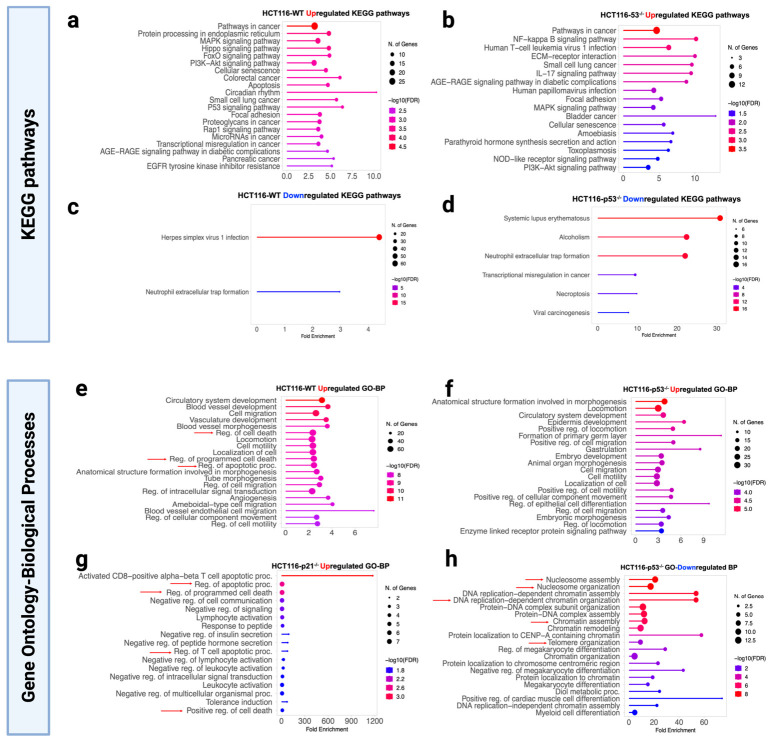
KEGG pathway and gene ontology (GO) enrichment analyses of differentially expressed genes in HCT116-WT and p53^−/−^ and p21^−/−^ cells following 4 mM metformin treatment for 48 h. The anged.significantly up- or down-regulated genes (FDR cutoff = 0.05) were analyzed on ShinyGO. (**a**) KEGG pathway enrichment of upregulated DEGs in WT. (**b**) KEGG pathway enrichment of upregulated DEGs in p53^−/−^ cells. (**c**) KEGG pathway enrichment of downregulated DEGs in WT. (**d**) KEGG pathway enrichment of downregulated DEGs in p53^−/−^ cells. (**e**) GO biological processes enrichment of upregulated DEGs in WT. (**f**) GO biological processes enrichment of upregulated DEGs in p53^−/−^ cells. (**g**) GO biological processes enrichment of upregulated DEGs in p21^−/−^ cells. (**h**) GO biological processes enrichment of downregulated DEGs in p53^−/−^ cells. Only statistically significant enrichments are shown. Red arrows highlight the selected pathways and biological processes of relevance.

Metformin-treated HCT116 wild-type (Figure 4a) showed enrichment of KEGG pathways related to cellular senescence and apoptotic signaling. Similarly, metformin-treated HCT116-p53^−/−^ cell line (Figure 4b) exhibited enrichment of the cellular senescence pathway, although fewer DEGs (Figure 4a,b) contributed to this pathway in the knockout cell line than in wild-type (6 vs. 10 DEGs; Figure 4a,b). Despite the smaller overall number of downregulated DEGs in p53^−/−^ cells (112 vs. 902 DEGs in WT; Figure 3c), several significant enriched pathways were identified among the downregulated genes (Figure 4c,d). In contrast, pathway enrichment analysis using ShinyGO indicated that the DEGs from the metformin-treated HCT116-p21^−/−^ cell line did not show statistically significant enrichment in KEGG pathways.

Commonly enriched pathways in both wild-type and p53^−/−^ cells included pathways in cancer, MAPK signaling pathway, cellular senescence, the AGE-RAGE signaling pathway in diabetic complications, and the PI3K-Akt signaling pathway (Figure 4a,b). However, the degree of enrichment varied between the two genotypes (Figure 4a,b). Neutrophil extracellular trap formation pathway was the only commonly downregulated pathway in both cell lines (Figure 4c,d).

### 3.6. Gene Ontology Analysis Highlighted the Differential Enrichment of Apoptotic and Chromatin-Related Processes Across Metformin-Treated HCT116 Cell Lines

Gene ontology analysis of the upregulated DEGs in metformin-treated HCT116-WT identified significant enrichment of biological processes related to regulation of cell death, programmed cell death, and apoptotic processes (Figure 4e). The metformin-treated HCT116-p53^−/−^ cell line demonstrated enrichment of biological processes associated with locomotion, positive regulation of cell migration, cell motility, and cell localization (Figure 4f). The metformin-treated HCT116-p21^−/−^ cell line exhibited enrichment of biological processes, similar to those observed in the wild-type, although with lower statistical significance (Figure 4g). Some of the overlapping biological processes in the two cell lines included the regulation of the apoptotic process and programmed cell death (Figure 4e,g). The most highly enriched GO term identified in p21^−/−^ cells was the “activated CD8-positive alpha-beta T cell apoptotic process” (Figure 4g). Given the small number of DEGs identified in p21^−/−^ cells, these enrichment results should be interpreted as exploratory. In contrast, gene ontology analysis of the downregulated genes in p21^−/−^ did not reveal any significant associations. GO analysis revealed that only the HCT116-p53^−/−^ cell line exhibited significant enrichment of downregulated DEGs, specifically for the chromatin-related biological processes, including nucleosome assembly, DNA replication-dependent chromatin assembly, DNA replication-dependent chromatin organization, protein-DNA complex subunit organization, chromatin assembly, chromatin remodeling, and telomere and chromatin organization (Figure 4h and Table 1). These enriched chromatin-related processes were not observed in the wild-type or p21^−/−^ cell lines.

The observed enrichment of chromatin-related biological processes in the p53-deficient cells is consistent with the established role of p53 in maintaining genome stability and regulating DNA repair and chromatin organization pathways [52]. In addition, cellular senescence was among the significantly enriched KEGG pathways following metformin treatment (Figure 4b). Collectively, these transcriptomic observations are consistent with senescence-associated transcriptional profile, including chromatin-related changes and reduced DNA metabolic activity, features commonly associated with transcriptionally repressive states [53].

## 4. Discussion

Metformin has emerged over the past decade as a promising repurposed drug in oncology, with numerous studies demonstrating its capacity to inhibit cancer cell growth [13,54,55,56]. However, as our results illustrate, its efficacy is not consistent across all genetic contexts. In this study, we examined how the presence or absence of two critical tumor suppressor genes, *TP53* and *CDKN1A/p21,* influences the anticancer effects of metformin in a *KRAS*-mutant CRC cell line. The presence of this mutation makes HCT116 a valuable in vitro model for investigating the biology of *KRAS*-driven tumors, which often exhibit limited responsiveness to currently available targeted therapies and therefore, require alternative therapeutic approaches [57]. Our findings provide insight into genotype-dependent responses to metformin in KRAS G13D-mutant CRC, a molecular subtype for which targeted therapeutic options remain limited, highlighting differences in phenotypic and transcriptomic responses associated with p53 and p21 status.

### 4.1. p53 and p21 Status in Relation to Metformin Response

We observed that wild-type HCT116 cells with functional p53 showed enrichment of cells in G0/G1 phase (Figure 2a–d) and upregulation of apoptosis-related biological processes (Figure 4a) following metformin treatment. The isogenic p53-deficient cells potentially also had a G1 arrest (Figure 2a–f), although this required further validation due to high variability observed in the data. These observations suggest that p53 status may influence cellular responses to metformin, consistent with the established role of p53 as the “guardian of the genome” in mediating cell-cycle arrest and cell death under metabolic stress conditions [52]. There is precedent in the literature that the antitumor effects of metformin often depend on the p53 status [58,59,60]. Our results align with those observations and suggest that p21 may contribute to the cellular response to metformin, consistent with its established role as a downstream effector of p53. We observed that in the absence of p21, the G1 arrest response to metformin was reduced (Figure 2), reflecting its established role as a cyclin-CDK inhibitor required for the G1 checkpoint enforcement [61]. Collectively, these findings suggest that p53 and p21 status may influence the cell-cycle response to metformin. Supporting this interpretation, transcriptomic analysis revealed a modest but statistically significant upregulation of *CDKN1A*, a canonical transcriptional target of p53, in metformin-treated wild-type cells (Table S2), suggesting engagement of the p53–p21 pathway in the transcriptional response to metformin.

Prior studies have also shown that metformin can still exert anticancer effects in p53-null cells and may sensitize them to additional therapies [62]. For example, metformin has been reported to radiosensitize p53-deficient colorectal cancer cells by inducing G2/M arrest and impairing DNA repair mechanisms [63]. While our data did not show consistent G2/M accumulation in the HCT116-p53^−/−^ cells, we observed variability in their cell-cycle response and a modest trend toward G0/G1 enrichment at 24 h (Figure 2a,e), accompanied by downregulation of genes involved in chromatin organization and DNA replication-associated processes (Figure 4h). These findings suggest that altered checkpoint regulation in p53-deficient cells may influence cellular responses to DNA damage and therapeutic stress. In contrast, p21-deficient cells displayed a distinct cell-cycle phenotype, characterized by increased accumulation in the G2/M phase following prolonged metformin exposure (Figure S1d). This pattern may reflect increased reliance on the G2/M checkpoint following loss of p21-mediated G1/S checkpoint control. Cancers with compromised G1 checkpoint function are known to depend more heavily on the G2/M checkpoint to maintain genomic stability under replication stress, making regulators of this checkpoint potential therapeutic targets [64]. *WEE1* is a key regulator of the G2/M checkpoint that delays mitotic entry to allow DNA repair [59]. In vitro studies using HCT116-p21^−/−^ cells have shown that inhibition of *WEE1* using adavosertib (AZD1775) increases replication-associated DNA damage and cell death, indicating that loss of p21 enhances vulnerability to checkpoint disruption [65]. Clinical evaluation of adavosertib in a randomized phase II FOCUS4-C trial demonstrated a significant improvement of progression-free survival in patients with *TP53*- and RAS-mutant metastatic colorectal cancer, supporting the therapeutic relevance of targeting *WEE1* in genetically defined colorectal cancer contexts [66], although its relevance to our experimental model requires direct validation.

### 4.2. Functional Interpretation of Common Metformin-Responsive Genes and Pathways

Among the common DEGs identified across the three cell lines (Figure 3d,e), one gene, *FAM111A* (Figure 3e), was consistently downregulated, whereas four genes, *DUSP5*, *FGD6*, *PLAUR*, and *TNFAIP3* (Figure 3d) were consistently upregulated. The known functions of these genes provide context for interpreting their shared responses to metformin. FAM111 trypsin-like peptidase A (*FAM111A*) is a serine protease that plays a significant role in DNA replication by facilitating the stable binding of DNA polymerase, loading the proliferating cell nuclear antigen (PCNA) onto the chromatin, and protecting replication forks from DNA-protein crosslinks, thereby maintaining genomic integrity [67,68]. Accordingly, the downregulation of *FAM111A* following metformin treatment may reflect altered DNA replication-associated processes. Among the four upregulated genes, dual-specificity phosphatase-5 (DUSP-5), an enzyme in the specific phosphatase family, regulates extracellular signal-regulated kinases 1 and 2 (ERK1/2) within the MAPK pathway [69]. The upregulation of *DUSP5* has the potential to influence downstream mitogen-activated protein kinase (MAPK) signaling and associated proliferative responses. Previous studies have reported *DUSP5* upregulation following metformin treatment [70], and an association between its expression and metformin IC50 [71], suggesting that *DUSP5* may contribute to the cellular response to metformin. The consistent upregulation of *FGD6* [72] and *PLAUR* [73], genes linked to cell migration and tumorigenesis, suggests that they may also contribute to the transcriptional changes associated with metformin treatment. *TNFAIP3,* a critical regulator of inflammation and an inhibitor of CRC cell proliferation and invasion, exhibited significant upregulation, which aligns with reports of its induction by metformin in other cancers [74].

The upregulation of *DUSP5* and downregulation of *FAM111A* are particularly noteworthy. *DUSP5* upregulation may reflect a negative feedback response within the MAPK pathway [69]. Since *KRAS* mutations strongly activate the MAPK/ERK cascade, upregulation of *DUSP5* may represent a negative-feedback response that partially counteracts pro-growth signals in *KRAS*-mutant cells. Meanwhile, the downregulation of *FAM111A* is consistent with altered DNA replication-associated processes. This may reflect reduced proliferative activity and perturbations of DNA replication. Whether these changes contribute to replication stress or genomic instability requires further investigation. Moreover, in wild-type cells, apoptosis-related biological processes were among the enriched pathways identified following metformin treatment (Figure 4a), consistent with a transcriptional response associated with cellular stress.

In p53^−/−^ cells, altered checkpoint regulation may influence cellular responses to replication stress. Previous studies have suggested that disruption of cell-cycle checkpoints can increase sensitivity to therapies targeting mitotic progression, including WEE1 inhibition [64], highlighting a potential area for future investigation. The consistent upregulation of *TNFAIP3* across all cell lines suggests that inflammatory signaling may contribute to the transcriptional response to metformin. *TNFAIP3* is frequently lost in certain lymphomas, leading to unchecked nuclear factor kappa-light-chain-enhancer of activated B cells (NF-κB) activity, which is a family of transcription factors that play a central role in regulating inflammatory and immune responses, survival, cell proliferation and apoptosis [75]. In our study, metformin was associated with increased *TNFAIP3* expression, which may be consistent with reduced NF-κB signaling. Chronic inflammation is a known promoter of colorectal tumorigenesis, and NF-κB drives expression of genes that inhibit apoptosis. Increased *TNFAIP3* expression may be associated with modulation of inflammatory signaling pathways. This observation is of interest in light of a recent study in pancreatic cancer patients undergoing upfront resection and metformin treatment [76]. The study reported significant alterations in immune profiles, specifically the reduced expression of pro-tumoral inflammation-related genes and the decreased macrophage infiltration as compared to non-metformin users [76]. These immune shifts were accompanied by an improved overall survival outcome, highlighting metformin’s potential role in modulating the tumor microenvironment and enhancing prognosis [76]. In colorectal cancer, it is plausible that metformin may influence immune surveillance through modulation of inflammatory signaling, potentially involving *TNFAIP3*, a possibility that warrants future in vivo investigation.

Another notable finding was the shared downregulation of the neutrophil extracellular trap (NET) formation pathway in metformin-treated wild-type and p53^−/−^ cells (Figure 4c,d). This observation is potentially relevant because NETs, complexes of unfolded DNA decorated with proteases, histones, cytosolic and granular proteins, have been implicated in cancer progression and metastatic behavior [77]. Proposed mechanisms include trapping circulating tumor cells [78], promoting epithelial–mesenchymal transition [79], and contributing to a tumor-supportive inflammatory microenvironment [80]. In colorectal cancer, metformin has been reported to suppress NET formation and to be associated with reduced tumor-associated neutrophils and NETs together with increased CD3+ and CD8+ tumor-infiltrating T cells. These observations support the biological relevance of the NET formation pathway as a potential downstream response to metformin exposure [80]. Since our study assessed transcriptomic pathway enrichment rather than NET formation directly, these findings should be interpreted cautiously.

## 5. Conclusions

Metformin treatment was associated with reduced cell viability and changes in cell-cycle distribution in *KRAS*-mutant HCT116 colorectal cancer cells, with these effects varying according to the status of the tumor suppressor genes p53 and p21. Wild-type cells exhibited the strongest transcriptional response following metformin treatment, including enrichment of biological processes related to cell-cycle regulation and apoptosis, whereas p53- and p21-deficient cells displayed more limited transcriptional and cell-cycle alterations. These findings suggest that phenotypic and transcriptomic responses to metformin may differ across genetic contexts, with tumor suppressor status potentially influencing treatment responsiveness. Although these findings were derived from an in vitro model, they provide a foundation for further investigation of genotype-dependent responses to metformin in colorectal cancer. Given metformin’s established safety profile and widespread clinical use, further functional validation and evaluation in additional in vivo models and genotype-stratified studies may help clarify its potential role as a repurposed therapeutic agent in colorectal cancer.

## Figures and Tables

**Table 1:** Gene ontology (GO) biological processes significantly enriched among differentially expressed genes in HCT116-WT and HCT116-p53^−/−^, HCT116-p21^−/−^ cell lines following treatment with 4 mM metformin for 48 h.

Direction	Adjusted *p*-Value	#Genes	Pathways
Down regulated	2.40 × 10^−3^	9	Nucleosome assembly
Down regulated	4.71 × 10^−3^	9	Chromatin assembly
Down regulated	9.14 × 10^−3^	9	Chromatin assembly or disassembly
Down regulated	9.14 × 10^−3^	9	Nucleosome organization
Up regulated	1.33 × 10^−6^	34	Locomotion
Up regulated	1.33 × 10^−6^	48	Animal organ development
Up regulated	1.80 × 10^−6^	18	Chemotaxis
Up regulated	1.80 × 10^−6^	29	Cell migration
Up regulated	1.80 × 10^−6^	18	Positive regulation of locomotion
Up regulated	1.80 × 10^−6^	18	Taxis
Up regulated	1.80 × 10^−6^	18	Positive regulation of cell motility
Up regulated	1.83 × 10^−6^	18	Positive regulation of cellular component movement
Up regulated	2.54 × 10^−6^	22	Regulation of cell migration
Up regulated	2.54 × 10^−6^	17	Positive regulation of cell migration
Up regulated	2.54 × 10^−6^	30	Cell motility
Up regulated	2.54 × 10^−6^	24	Regulation of cellular component movement
Up regulated	2.54 × 10^−6^	30	Localization of cell
Up regulated	2.95 × 10^−6^	23	Regulation of locomotion
Up regulated	6.21 × 10^−6^	34	Movement of cell or subcellular component

## Data Availability

The original contributions presented in this study are included in the article and Supplementary Material. Further inquiries can be directed to the corresponding authors.

## Supplementary Materials

The following supporting information can be downloaded at: https://www.mdpi.com/article/10.3390/cimb48070731/s1.

## Abbreviations

The following abbreviations are used in this manuscript:

CRC	Colorectal cancer
HCT116	Human Colon Tumor cells
TP53	Tumor Protein p53
CDKN1A	Cyclin-Dependent Kinase Inhibitor 1A
KRAS	Kristen Rat Sarcoma Proto-oncogene
G0/G1	Gap 0 phase/Growth 1 phase
G13D	Glycine (G) & Aspartic Acid (D) at position 13 of the protein
G12C	Glycine (G) & Cysteine (C) at position 12 of the protein
WT	Wild-type
KO	Knockout
DEG	Differential Expressed Genes
MAPK	Mitogen-Activated Protein Kinase
APC	Adenomatous Polyposis Coli
IGF-1	Insulin-like Growth Factor 1
AMPK	AMP-activated Protein Kinase
mTOR	Mammalian Target of the Rapamycin
CSCs	Cancer Stem Cells
RTK	Receptor Tyrosine Kinase
EGFR	Epidermal Growth Factor Receptor
GTP	Guanosine Triphosphate
ROS	Reactive Oxygen Species
PI3K-Akt	Phosphoinositide-3-kinase/Protein Kinase B
ERK	Extracellular Signal-regulated Kinase
ATCC	American Type Culture Collection
DMEM	Dulbecco’s Modified Eagle Medium
CO_2_	Carbon Dioxide
RIPA	RadioImmunoprecipitation Assay Buffer
DTT	Dithiothreitol
PMSF	Phenylmethylsulfonyl Fluoride
SDS	Sodium Dodecyl Sulfate
Tris-HCl	Tris(hydroxymethyl)aminomethane Hydrochloride
pH	Potential of Hydrogen
TBST	Tris-Buffered Saline with Tween-20
BSA	Bovine Serum Albumin
Ser15	Serine 15
Waf1/Cip1	Wild-type p53-Activated Fragment 1/CDK-Interacting Protein 1
HRP	Horseradish Peroxidase
ECL	Enhanced Chemiluminescence
MTT	3-(4,5-dimethylthiazol-2-yl)-2,5-diphenyltetrazolium Bromide
PBS	Phosphate-Buffered Saline
PI	Propidium Iodide
RNase A	Ribonuclease A
DNA	Deoxyribonucleic Acid
RNA	Ribonucleic Acid
PCA	Principal Component Analysis
GO	Gene Ontology
RT-qPCR	Quantitative Real-Time Polymerase Chain Reaction
KEGG	Kyoto Encyclopedia of Genes and Genomes
G2/M	Gap 2 phase/Mitosis
ANNOVA	Analysis of Variance
PC1	Principal Component 1
PC2	Principal Component 2
G1	Growth 1 phase
DUSP5	Dual Specificity Phosphatase 5
FGD6	FYVE, RhoGEF and PH domain containing 6
PLAUR	Plasminogen Activator, Urokinase Receptor
TNFAIP3	Tumor Necrosis Factor Alpha-Induced Protein 3
FAM111A	FAM111 Trypsin-like Peptidase A
AGE-RAGE	Advanced Glycation End-products–Receptor for Advanced Glycation End-products
CD8	Cluster of Differentiation 8
G1/S	Gap 1 phase/Synthesis
WEE1	WEE1-like Protein Kinase
PCNA	Proliferating Cell Nuclear Antigen
IC50	Half-Maximal Inhibitory Concentration
NF-κB	Nuclear Factor kappa-light-chain-enhancer of Activated B cells
NETs	Neutrophil Extracellular Trap
CD3	Cluster of Differentiation 3
RIN	RNA Integrity Number
FDR	False Discovery Rate
SD	Standard Deviation
CV	Coefficient of Variation
