# Supplementary material for: p53 and p21 Status Influences Cellular Response to Metformin in KRAS-Mutant HCT116 Colorectal Cancer Cells
Source: Curr Issues Mol Biol. 2026 Jul 17;48(7):731. doi: 10.3390/cimb48070731 (PMC13408822; doi:10.3390/cimb48070731)
Supplement: Supplementary file 1 [file cimb-48-00731-s001.zip › Supplementary_Figures.pdf]

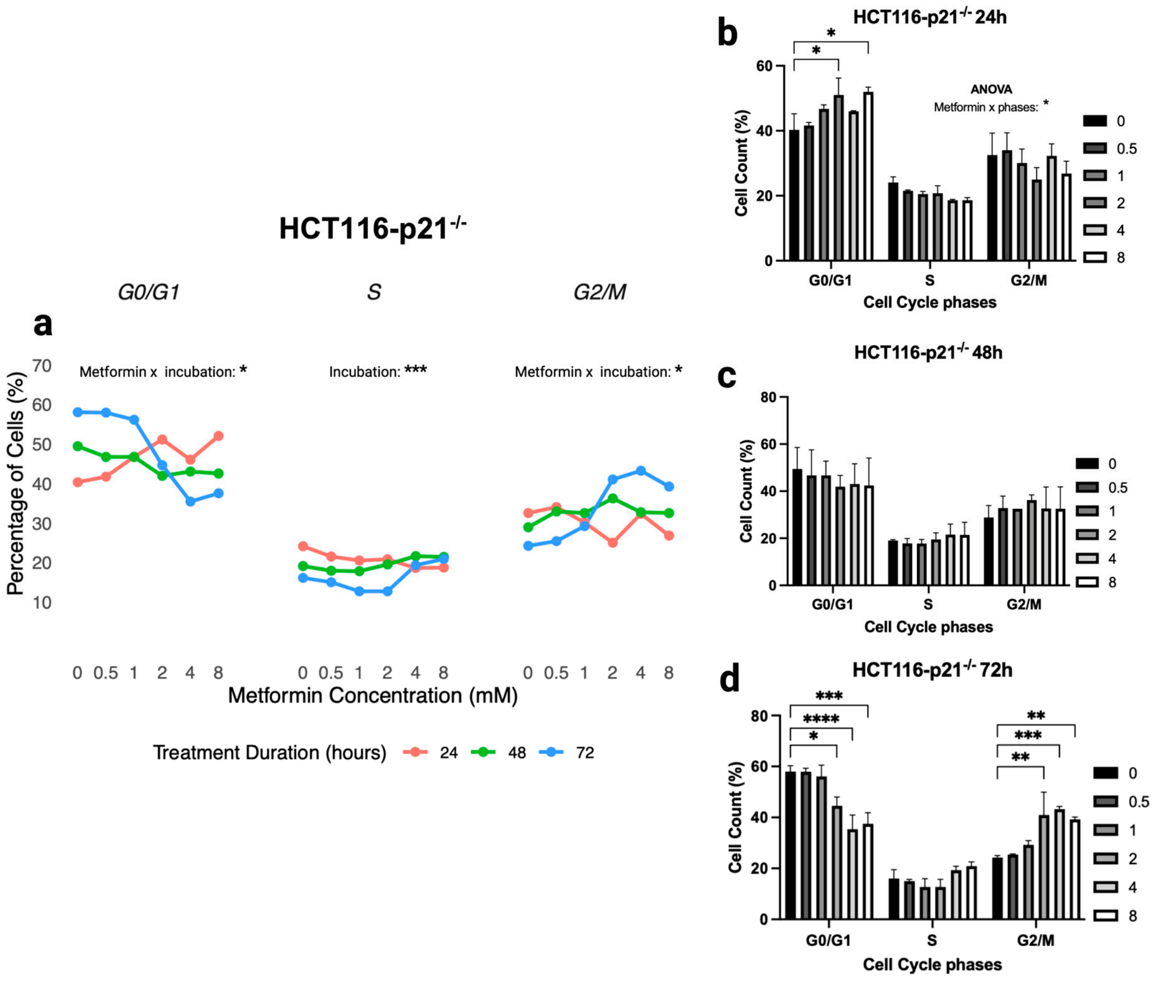

Figure S1. Cell cycle analysis by propidium iodide staining in HCT116-p21$^{-/-}$ treated with 0-8 mM of metformin for 24, 48 and 72 hours. (a) Cell cycle phase trends across the three incubation periods. (b) HCT116-p21$^{-/-}$ 24h treatment bar plot. (c) HCT116-p21$^{-/-}$ 48h treatment bar plot (d) HCT116-p21$^{-/-}$ 72h treatment bar plot. Two-way ANOVA was performed using GraphPad Prism with Tukey's multiple comparison test (n = 2), *p = 0.0332; **p = 0.0021; ***p = 0.0002; ****p <0.0001 versus untreated control.

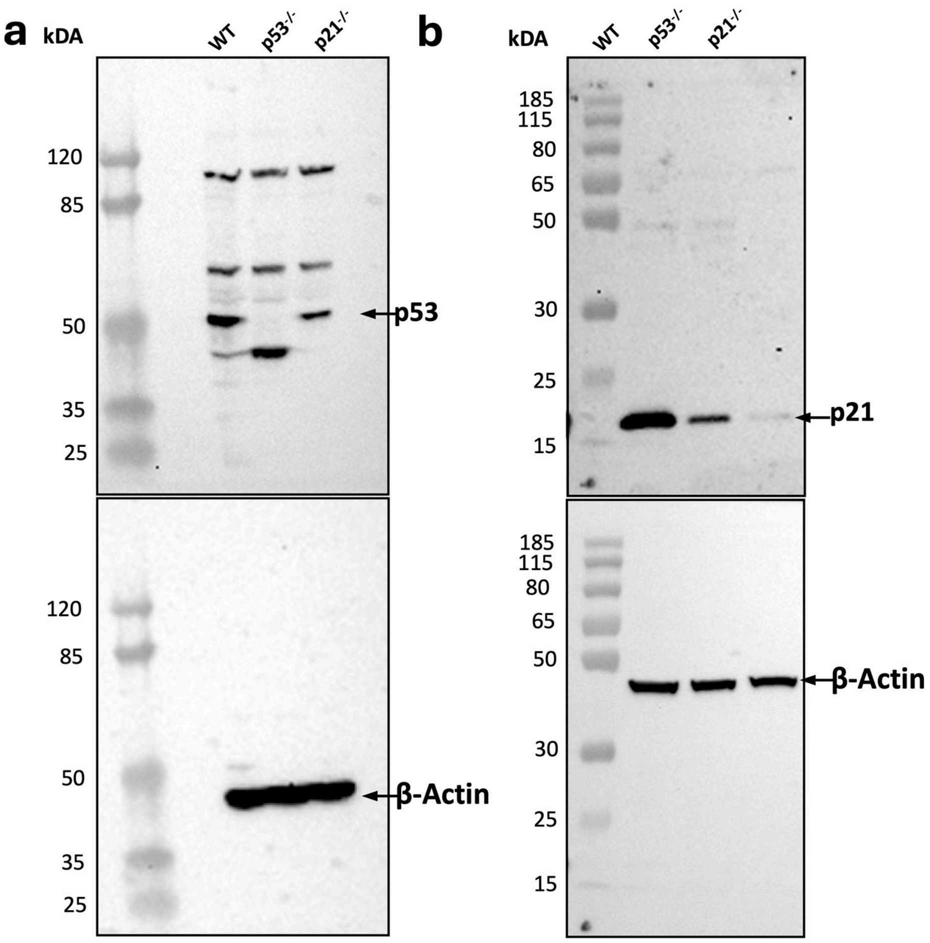

Figure S2: Western blot validation of p53 and p21 knockout status in HCT116 derivative cell lines under untreated conditions. (a) Detection of phospho-p53 (Ser15) and β-actin. A phospho-p53 (Ser15) signal at the expected molecular weight was observed in the wild-type and p21$^{-/-}$ cells but not in p53$^{-/-}$ cells. (b) Detection of p21 and the β-actin. The p21 protein was detected in both wild-type and p53$^{-/-}$ cells but was negligible in p21$^{-/-}$ cells. The β-actin was used as a loading control. Molecular weight markers (kDa) are indicated on left side of the blots.

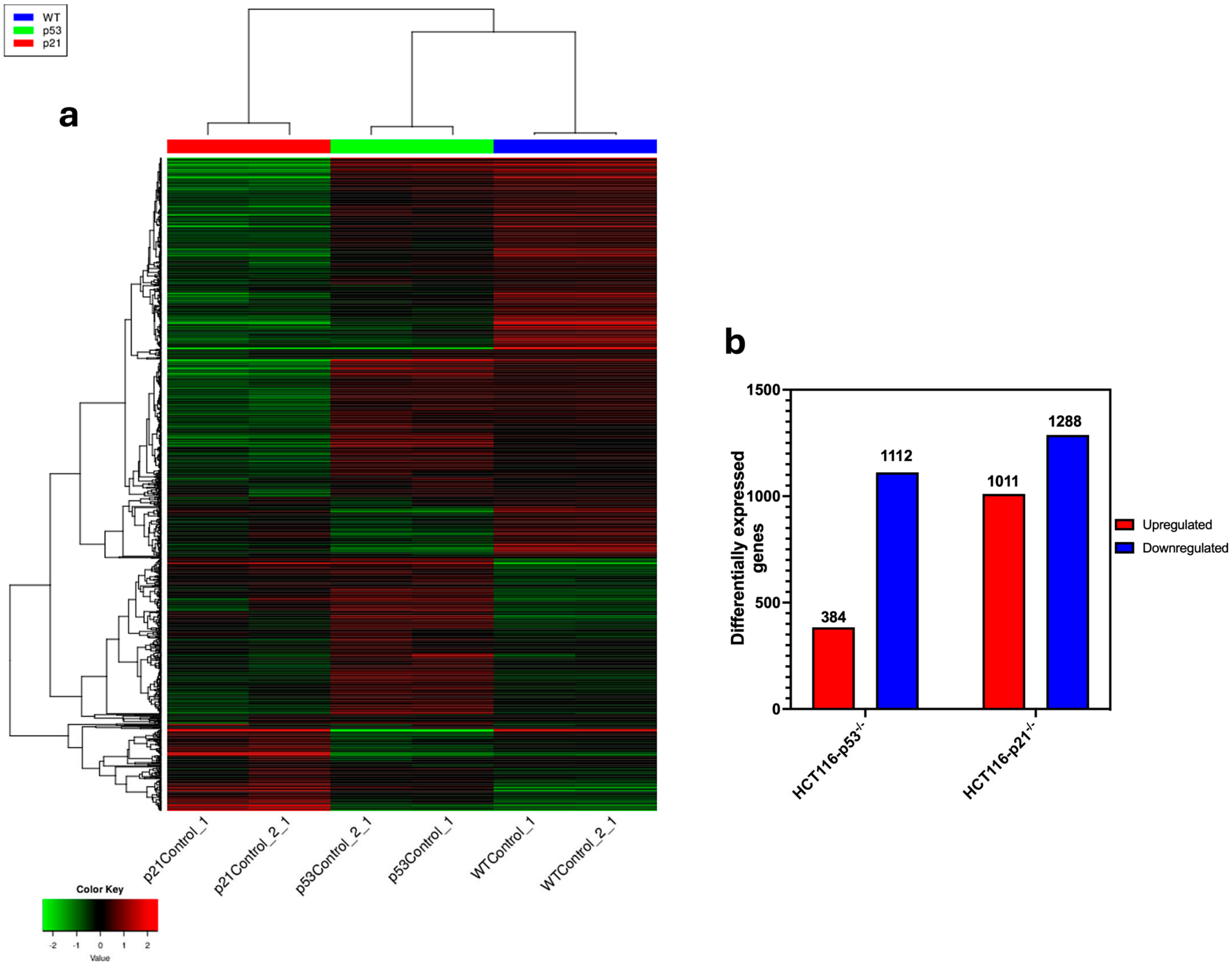

Figure S3. Transcriptomic analysis of untreated HCT116-WT, HCT116-p53$^{-/-}$ and HCT116-p21$^{-/-}$ cells (n = 2) at FDR cutoff of 0.05. (a) Heatmap of differential expression analysis of untreated samples relative to untreated HCT116-WT. (b) Number of differentially expressed genes in untreated HCT116-p53$^{-/-}$ and HCT116-p21$^{-/-}$ relative to untreated HCT116-WT cells.

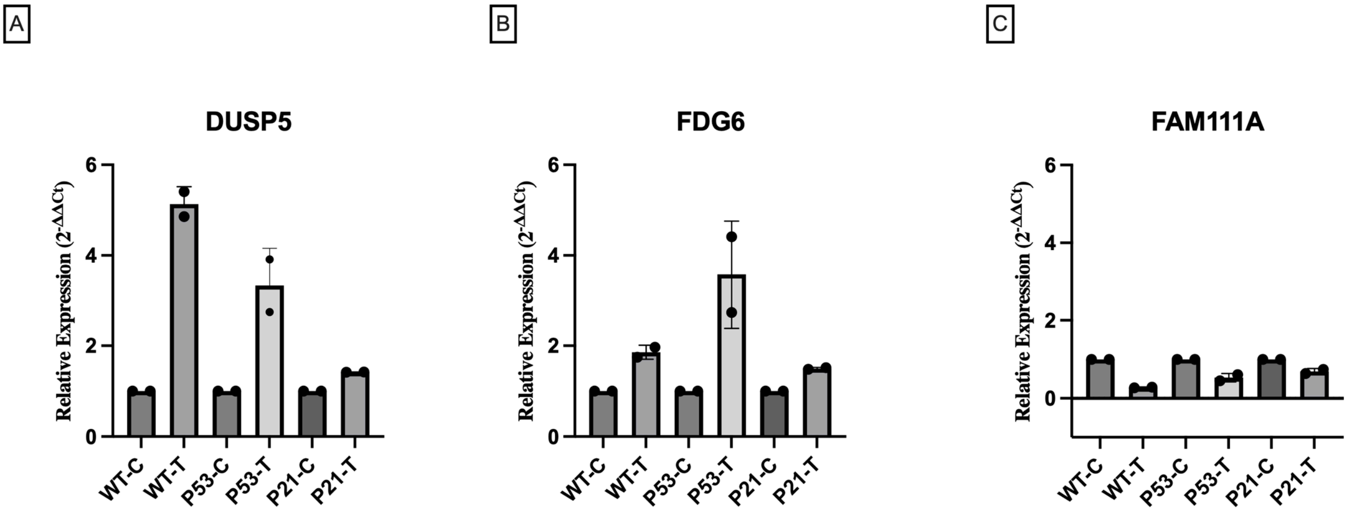

Figure S4: Quantitative Real-Time PCR validation of metformin-responsive genes identified by RNA sequencing. Relative mRNA expression of (A) *DUSP5*, (B) *FGD6*, and (C) *FAM111A* in HCT116 wild-type (WT), $p53^{-/-}$, and $p21^{-/-}$ cells following treatment with 4 mM metformin for 48 h. Gene expression was measured by RT-qPCR using β-actin as the endogenous reference gene and calculated using the $2^{-\Delta\Delta Ct}$ method. Expression levels were normalized to the corresponding untreated control for each genotype (WT-C, p53-C, and p21-C), which were set to 1. Data points represent two independent biological replicates, and bars indicate the mean ± SD. *DUSP5* and *FGD6* were upregulated, whereas *FAM111A* was downregulated following metformin treatment across all three genotypes, consistent with the RNA-seq findings.

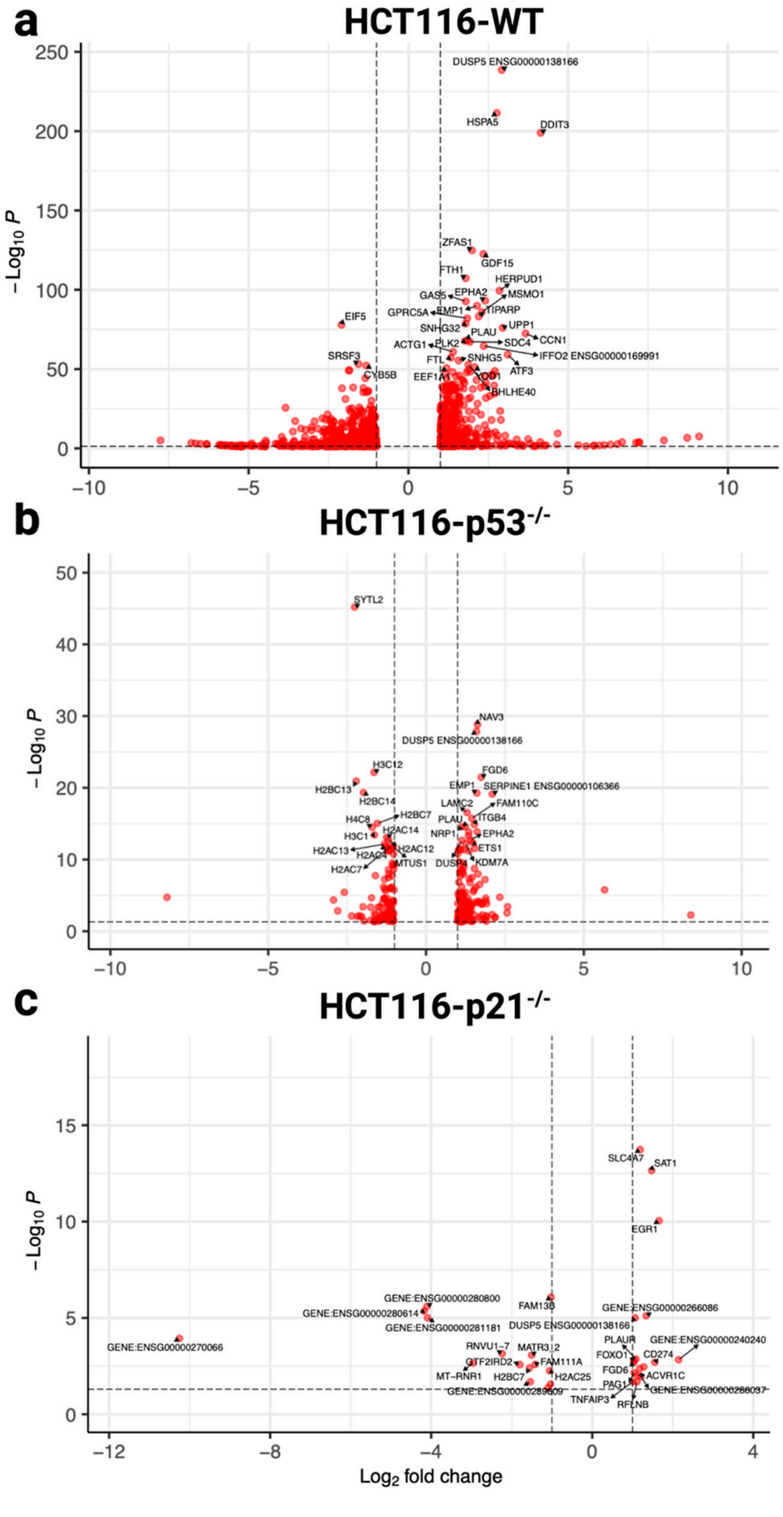

Figure S5. Volcano plots of significant DEGs following metformin treatment (4 mM, 48h). (a) HCT116 wild-type with 1399 DEGs. (b) HCT116-p53$^{-/-}$ with 270 DEGs. (c) HCT116-p21$^{-/-}$ with 32 DEGs. Red circles represent genes meeting the significance threshold (adjusted $p < 0.05$ and $|\log2 \text{ fold change}| \geq 1$).
